# Supplementary material for: Metagenomic DNA sequencing to quantify Mycobacterium tuberculosis DNA and diagnose tuberculosis
Source: Sci Rep. 2022 Oct 10;12:16972. doi: 10.1038/s41598-022-21244-x (PMC9551046; doi:10.1038/s41598-022-21244-x)
Supplement: Supplementary file 3 — Supplementary Information 3. [file 41598_2022_21244_MOESM3_ESM.docx]

**Supplemental Information for:**

**Metagenomic DNA sequencing to quantify *Mycobacterium tuberculosis* DNA and diagnose tuberculosis**

Adrienne Chang^1,^ Omary Mzava^1,^ Liz-Audrey Kounatse Djomnang^1,^ Joan Sesing Lenz^1,^ Philip Burnham^1,^ Peter Kaplinsky^1,^ Alfred Andama^2,^ John Connelly^3,^ Christine M. Bachman^3,^ Adithya Cattamanchi^4,^ Amy Steadman^3,^ Iwijn De Vlaminck^1*^

**Affiliations:**

^1^Nancy E. and Peter C. Meinig School of Biomedical Engineering, Cornell University, Ithaca, New York, USA

^2^ Department of Medical Microbiology, Makerere University, Kampala, Uganda

^3^Global Health Labs, Bellevue, Washington, US

^4^Center for Tuberculosis and Division of Pulmonary and Critical Care Medicine, University of California San Francisco, San Francisco, California, USA

*Corresponding author at: vlaminck@cornell.edu

**Table S1.** Diagnostic performance of *M. tuberculosis* cfDNA using a limit of detection of 0.1 RPM.

| **Biofluid** | **Group** | **Sensitivity** | **Specificity** |
| --- | --- | --- | --- |
| Plasma | Sputum Positive vs Sputum Negative | 88% (15/17) | 4.5% (2/44) |
|  | Tuberculosis vs Endemic | 93% (57/61) | 6.6% (4/61) |
|  | Tuberculosis vs Non-endemic | 97% (57/59) | 95% (37/39) |
| Urine | Sputum Positive vs Sputum Negative | 97% (29/30) | 7.1% (2/28) |
|  | Tuberculosis vs Endemic | 94% (45/48) | 30% (7/23) |
|  | Tuberculosis vs Non-endemic | 94% (45/48) | 63% (89/141) |
| Oral Swab | Sputum Positive vs Sputum Negative | 100% (27/27) | 0% (0/15) |
|  | Tuberculosis vs Non-endemic | 100% (42/42) | 0% (0/3) |

**Table S2.** Diagnostic performance of *M. tuberculosis* cfDNA

| **Group** | **Biofluid** | **Group Size** | **Area Under the Curve** | **95% Confidence Interval** | **Figure** |
| --- | --- | --- | --- | --- | --- |
| Sputum Positive vs Sputum negative | Plasma | Sputum Negative n=44  Sputum Positive n=17 | 0.6023 | 0.4417-0.7629 | 3A |
|  | Urine | Sputum Negative n=28  Sputum Positive n=30 | 0.5964 | 0.4477-0.7451 |  |
|  | Oral Swab | Sputum Negative n=15  Sputum Positive n=27 | 0.5012 | 0.3165-0.686 |  |
| Tuberculosis vs Endemic | Plasma | Tuberculosis n=61  Endemic n=61 | 0.64985 | 0.552-0.7476 | 3B |
|  | Urine | Tuberculosis n=58 Endemic n=23 | 0.6151 | 0.4394-0.7194 |  |
| Tuberculosis vs Non-endemic | Plasma | Tuberculosis n=61  Non-endemic n=39 | 0.9697 | 0.9385-1 | 3C |
|  | Urine | Tuberculosis n=58  Non-endemic n=141 | 0.9265 | 0.8598-0.9594 |  |
|  | Oral Swab | Tuberculosis n=42  Non-endemic n=3 | 0.9921 | 0.9701-1 |  |

**Table S3.** Description of databases tested for metagenomic classification. The genome assemblies are provided in Supplementary Data 1.

| **Name** | **Description** |
| --- | --- |
| MYCO2015 | All *Mycobacterium* complete genomes submitted after 2015 |
| MYCO_REF | All *Mycobacterium* reference genomes |
| TB2015 | All *M. tuberculosis* complete genomes submitted after 2015 |
| TB_REF | *M. tuberculosis* *H37Rv* reference genome |
| NTM_PD | Pulmonary disease-causing *Mycobacterium* reference genomes |
| NTM_ALL | Disease-causing *Mycobacterium* reference genomes |
| BAC_MYCO | All *Mycobacterium* reference genomes identified by RefSeq and the Pathosystems Resource Integration Center (PATRIC) pathogenic reference bacterial genomes |
| BAC_TB | *M. tuberculosis H37Rv* reference genome and the PATRIC pathogenic reference bacterial genomes |
| BAC_NTM | Non-tuberculous *Mycobacterium* and the PATRIC pathogenic reference bacterial genomes |
| BAC_PD | Pulmonary disease-causing *Mycobacterium* reference genomes and the PATRIC pathogenic reference bacterial genomes |

**Table S4.** Diagnostic performance summary of the 10 different databases with and without removal of confounding ribosomal RNA sequences for urine and plasma given as area under the curve values.

| **Database** | **Plasma**  **Sputum positive vs Sputum negative** | | **Plasma**  **Sputum positive vs Non-endemic** | | **Urine**  **Sputum positive vs Sputum negative** | | **Urine**  **Sputum positive vs Non-Endemic** | |
| --- | --- | --- | --- | --- | --- | --- | --- | --- |
|  | **All** | **-rRNA** | **All** | **-rRNA** | **All** | **-rRNA** | **All** | **-rRNA** |
| **TB2015** | 0.59 | 0.6 | 0.82 | 0.79 | 0.62 | 0.62 | 0.81 | 0.82 |
| **TB_REF** | 0.72 | 0.7 | 1 | 0.96 | 0.65 | 0.63 | 0.84 | 0.91 |
| **NTM_PD** | 0.72 | 0.68 | 1 | 0.96 | 0.65 | 0.65 | 0.85 | 0.91 |
| **NTM_ALL** | 0.72 | 0.69 | 1 | 0.97 | 0.63 | 0.62 | 0.84 | 0.91 |
| **MYCO2015** | 0.62 | 0.62 | 0.3 | 0.33 | 0.61 | 0.6 | 0.8 | 0.79 |
| **MYCO_REF** | 0.63 | 0.61 | 0.97 | 0.97 | 0.61 | 0.6 | 0.91 | 0.93 |
| **BAC_TB** | 0.71 | 0.69 | 1 | 0.96 | 0.62 | 0.61 | 0.91 | 0.93 |
| **BAC_PD** | 0.7 | 0.67 | 1 | 0.96 | 0.62 | 0.62 | 0.91 | 0.92 |
| **BAC_NTM** | 0.7 | 0.68 | 0.99 | 0.97 | 0.62 | 0.61 | 0.91 | 0.93 |
| **BAC_MYCO** | 0.62 | 0.6 | 0.97 | 0.97 | 0.6 | 0.59 | 0.94 | 0.94 |

**Table S5.** Overview of diagnostic performance of published nucleic acid assay for tuberculosis diagnostics in plasma and urine (NR = not reported).

| **Author** | **Year** | **Biofluid** | **Target** | **Amplicon Length** | **Sensitivity** | **Specificity** | **Location** | **Cohort** |
| --- | --- | --- | --- | --- | --- | --- | --- | --- |
| Patel[^1^](https://www.zotero.org/google-docs/?jNAA4X) | 2018 | Urine | DR region | 38 bp | 42.90% | 88.60% | South Africa | 175 Culture Positive |
|  |  |  |  |  |  |  |  | 238 Culture Negative |
| Ushio[^2^](https://www.zotero.org/google-docs/?EqeF3q) | 2016 | Plasma | IS6110 | 71 bp | 65% | 93% | Japan | 24 Culture Positive |
|  |  |  |  |  |  |  |  | 15 Healthy |
| Ushio[^2^](https://www.zotero.org/google-docs/?Xp8NNa) | 2016 | Plasma | gyrB | 137 bp | 29% | 100% | Japan | 24 Culture Positive |
|  |  |  |  |  |  |  |  | 15 Healthy |
| Click[^3^](https://www.zotero.org/google-docs/?qYPIPU) | 2018 | Plasma | IS6110 | 106 bp | 45% | 67% | Kenya | 47 Culture Positive |
|  |  |  |  |  |  |  |  | 3 Culture Negative |
| Cannas[^4^](https://www.zotero.org/google-docs/?PZeaVn) | 2008 | Urine | IS6110 | 67 bp | 79% | 100% | Italy | 43 Culture Positive |
|  |  |  |  |  |  |  |  | 10 Culture Negative |
|  |  |  |  |  |  |  |  | 13 Healthy |
| Fortun[^5^](https://www.zotero.org/google-docs/?qVmHJt) | 2014 | Urine | 16S-rRNA | NR | 17.90% | NR | Spain | 28 Culture Positive |
| Labugger[^6^](https://www.zotero.org/google-docs/?WwuZW5) | 2017 | Urine | IS6110 | 38 bp | 64% | 100% | Germany | 11 Culture Positive |
|  |  |  |  |  |  |  |  | 8 Culture Negative |
| Torrea[^7^](https://www.zotero.org/google-docs/?l5gVOn) | 2006 | Urine | IS6110 | NR | 40.60% | 98.20% | Burkina Faso | 210 Culture Positive |
|  |  |  |  |  |  |  |  | 55 Culture Negative |
| Rebollo[^8^](https://www.zotero.org/google-docs/?P7CST8) | 2006 | Urine | IS6110 | 123 bp | 42% | 100% | Spain | 43 Culture Positive |
|  |  |  |  |  |  |  |  | 14 Culture Negative |
|  |  |  |  |  |  |  |  | 13 Healthy |
|  |  |  |  |  |  |  |  | 13 Other Disease |
| Kafwabulula[^9^](https://www.zotero.org/google-docs/?4nP011) | 2002 | Urine | NR | NR | 55.60% | 98.40% | Zambia | 63 Culture Positive |
|  |  |  |  |  |  |  |  | 63 Culture Negative |
| Oreskovic[^10^](https://www.zotero.org/google-docs/?KR2zBC) | 2020 | Urine | IS6110 | 40 bp | 83.70% | 100% | South Africa | 49 Culture Positive |
|  |  |  |  |  |  |  |  | 10 Culture Negative |
|  |  |  |  |  |  |  |  | 14 Healthy Non-Endemic |

**Table S6.** Classification of synthetic *M. tuberculosis* reads at varying read coverages in urine samples from non-endemic (n=20, 3 replicates per sample) and tuberculosis (n=20, 3 replicates per sample) cohorts after removal of either host or host and *Mycobacterium* reads.

| **Cohort** | **Data** | **N** | **# Simulated** | **# Mapped** | **Accuracy** | **Precision** | **Sensitivity** | **F1** |
| --- | --- | --- | --- | --- | --- | --- | --- | --- |
| Non-endemic | Non-host | 60 | 2.69 | 2.69 | 0.977 | 0.844 | 0.977 | 0.889 |
| Non-endemic | Non-host | 60 | 5.86 | 5.78 | 0.981 | 0.912 | 0.987 | 0.939 |
| Non-endemic | Non-host | 60 | 13.6 | 13.4 | 0.98 | 0.953 | 0.988 | 0.968 |
| Non-endemic | Non-host | 60 | 158 | 156 | 0.974 | 0.975 | 0.992 | 0.982 |
| Non-endemic | Non-host, Non-*Mycobacterium* | 60 | 2.69 | 2.67 | 0.95 | 0.966 | 0.966 | 0.966 |
| Non-endemic | Non-host, Non-*Mycobacterium* | 60 | 5.86 | 5.83 | 0.978 | 1 | 0.994 | 0.997 |
| Non-endemic | Non-host, Non-*Mycobacterium* | 60 | 13.6 | 13.5 | 0.977 | 1 | 0.993 | 0.995 |
| Non-endemic | Non-host, Non-*Mycobacterium* | 60 | 158 | 156 | 0.973 | 1 | 0.995 | 0.997 |
| Tuberculosis | Non-host | 60 | 16.9 | 16.7 | 0.972 | 0.216 | 0.988 | 0.309 |
| Tuberculosis | Non-host | 60 | 54.5 | 53.9 | 0.972 | 0.375 | 0.986 | 0.491 |
| Tuberculosis | Non-host | 60 | 173 | 171 | 0.975 | 0.799 | 0.989 | 0.857 |
| Tuberculosis | Non-host | 60 | 888 | 878 | 0.975 | 0.799 | 0.989 | 0.857 |
| Tuberculosis | Non-host, Non-*Mycobacterium* | 60 | 16.9 | 16. | 0.974 | 0.995 | 0.984 | 0.989 |
| Tuberculosis | Non-host, Non-*Mycobacterium* | 60 | 54.5 | 53.9 | 0.973 | 1 | 0.988 | 0.994 |
| Tuberculosis | Non-host, Non-*Mycobacterium* | 60 | 173 | 171 | 0.977 | 1 | 0.986 | 0.993 |
| Tuberculosis | Non-host, Non-*Mycobacterium* | 60 | 888 | 878 | 0.976 | 1 | 0.989 | 0.994 |

**Table S7.** Classification of synthetic *M. bovis* reads at varying read coverages in urine samples from non-endemic (n=20, 3 replicates per sample) and tuberculosis (n=20, 3 replicates per sample) cohorts after removal of either host or host and *Mycobacterium* reads.

| **Cohort** | **Data** | **N** | **# Simulated** | **# Mapped** | **Accuracy** | **Precision** | **Sensitivity** | **F1** |
| --- | --- | --- | --- | --- | --- | --- | --- | --- |
| Non-endemic | Non-host | 60 | 2.69 | 2.69 | 0.989 | 0.827 | 0.98 | 0.88 |
| Non-endemic | Non-host | 60 | 5.86 | 5.78 | 1 | 0.903 | 0.99 | 0.938 |
| Non-endemic | Non-host | 60 | 13.6 | 13.4 | 1 | 0.951 | 0.987 | 0.967 |
| Non-endemic | Non-host | 60 | 158 | 156 | 1 | 0.976 | 0.979 | 0.975 |
| Non-endemic | Non-host, Non-*Mycobacterium* | 60 | 2.69 | 2.67 | 1 | 0.948 | 1 | 0.97 |
| Non-endemic | Non-host, Non-*Mycobacterium* | 60 | 5.86 | 5.83 | 0.997 | 0.974 | 0.981 | 0.974 |
| Non-endemic | Non-host, Non-*Mycobacterium* | 60 | 13.6 | 13.5 | 0.999 | 0.989 | 0.99 | 0.989 |
| Non-endemic | Non-host, Non-*Mycobacterium* | 60 | 158 | 156 | 0.999 | 0.995 | 0.992 | 0.989 |
| Tuberculosis | Non-host | 60 | 16.9 | 16.7 | 1 | 0.199 | 0.985 | 0.28 |
| Tuberculosis | Non-host | 60 | 54.5 | 53.9 | 1 | 0.359 | 0.989 | 0.478 |
| Tuberculosis | Non-host | 60 | 173 | 171 | 1 | 0.566 | 0.988 | 0.675 |
| Tuberculosis | Non-host | 60 | 888 | 878 | 1 | 0.796 | 0.989 | 0.856 |
| Tuberculosis | Non-host, Non-*Mycobacterium* | 60 | 16.9 | 16. | 0.999 | 1 | 0.993 | 0.996 |
| Tuberculosis | Non-host, Non-*Mycobacterium* | 60 | 54.5 | 53.9 | 1 | 1 | 0.991 | 0.995 |
| Tuberculosis | Non-host, Non-*Mycobacterium* | 60 | 173 | 171 | 1 | 1 | 0.988 | 0.994 |
| Tuberculosis | Non-host, Non-*Mycobacterium* | 60 | 888 | 878 | 1 | 1 | 0.989 | 0.994 |

**Table S8.** Classification of synthetic *M. avium* reads at varying read coverages in urine samples from non-endemic (n=20, 3 replicates per sample) and tuberculosis (n=20, 3 replicates per sample) cohorts after removal of either host or host and *Mycobacterium* reads.

| **Cohort** | **Data** | **N** | **# Simulated** | **# Mapped** | **Accuracy** | **Precision** | **Sensitivity** | **F1** |
| --- | --- | --- | --- | --- | --- | --- | --- | --- |
| Non-endemic | Non-host | 60 | 2.69 | 2.69 | 0.971 | 0.629 | 0.966 | 0.713 |
| Non-endemic | Non-host | 60 | 5.86 | 5.78 | 0.98 | 0.745 | 0.968 | 0.818 |
| Non-endemic | Non-host | 60 | 13.6 | 13.4 | 0.976 | 0.846 | 0.941 | 0.882 |
| Non-endemic | Non-host | 60 | 158 | 156 | 0.983 | 0.924 | 0.934 | 0.922 |
| Non-endemic | Non-host, Non-*Mycobacterium* | 60 | 2.69 | 2.67 | 0.95 | 0.761 | 0.929 | 0.823 |
| Non-endemic | Non-host, Non-*Mycobacterium* | 60 | 5.86 | 5.83 | 0.947 | 0.852 | 0.912 | 0.869 |
| Non-endemic | Non-host, Non-*Mycobacterium* | 60 | 13.6 | 13.5 | 0.973 | 0.94 | 0.946 | 0.937 |
| Non-endemic | Non-host, Non-*Mycobacterium* | 60 | 158 | 156 | 0.98 | 0.877 | 0.947 | 0.908 |
| Tuberculosis | Non-host | 60 | 16.9 | 16.7 | 0.981 | 0.197 | 0.946 | 0.292 |
| Tuberculosis | Non-host | 60 | 54.5 | 53.9 | 0.979 | 0.366 | 0.951 | 0.503 |
| Tuberculosis | Non-host | 60 | 173 | 171 | 0.981 | 0.621 | 0.946 | 0.74 |
| Tuberculosis | Non-host | 60 | 888 | 878 | 0.98 | 0.877 | 0.947 | 0.908 |
| Tuberculosis | Non-host, Non-*Mycobacterium* | 60 | 16.9 | 16. | 0.973 | 0.99 | 0.928 | 0.954 |
| Tuberculosis | Non-host, Non-*Mycobacterium* | 60 | 54.5 | 53.9 | 0.977 | 1 | 0.943 | 0.969 |
| Tuberculosis | Non-host, Non-*Mycobacterium* | 60 | 173 | 171 | 0.981 | 1 | 0.946 | 0.972 |
| Tuberculosis | Non-host, Non-*Mycobacterium* | 60 | 888 | 878 | 0.981 | 1 | 0.946 | 0.972 |

**Table S9.** Insertion sequences used to classify the nontuberculous mycobacterial background.

| **Species** | **Insertion sequences** | **Accession** |
| --- | --- | --- |
| *Mycobacterium avium* | IS1110 | Z23003.1 |
|  | IS1245 | L33879 |
|  | IS1311 | CP000479 |
|  | IS1601 | CP000479 |
|  | IS1612 | AJ251813 |
|  | IS902 | X58030 |
|  | IS666 | AF107207 |
|  | IS999 | AF232829 |
| *Mycobacterium branderi* | IS1408 | U62766 |
| *Mycobacterium celatum* | IS1407 | X97307 |
| *Mycobacterium fortuitum* | IS219 | MF018875 |
|  | IS220 | AJ315500 |
| *Mycobacterium gordonae* | IS1511 | U95315 |
|  | IS1512 | U95314 |
| *Mycobacterium intracellulare* | IS1141 | L10239 |
| *Mycobacterium smegmatis* | IS1096 | M76495 |
|  | IS1137 | X70913 |
|  | IS1549 | CP000480 |
|  | IS6120 | M69182 |
| *Mycobacterium ulcerans* | IS2404 | AF003002 |
| *Mycobacterium xenopi* | IS1395 | U35051 |


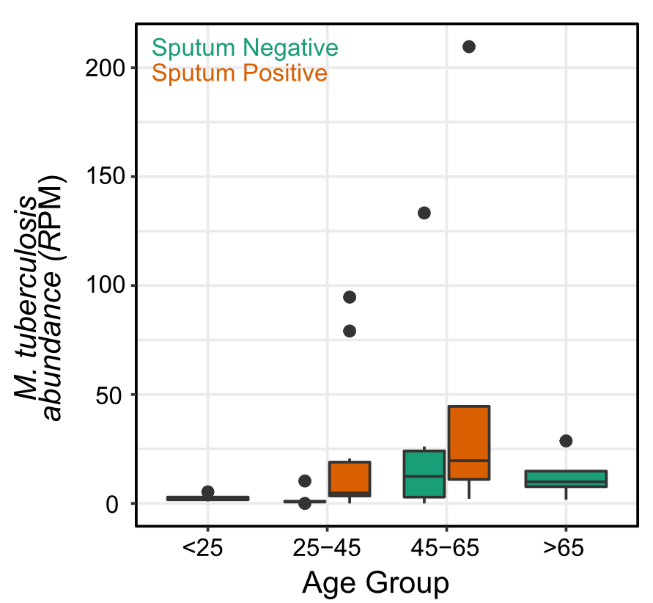


**Figure S1.** Correlations between age and *M. tuberculosis* abundance.

**Description of Additional Supplementary Files**

File Name: Supplementary Data 1 (SuppData1.xslx)

Description: Genome assemblies used to create the different databases used for metagenomic classification.

File Name: Supplementary Data 2 (SuppData2.xslx)
Description: Number of TB reads detected in each sample

**References**

[1. Patel, K. *et al.* Evaluation of a Urine-Based Rapid Molecular Diagnostic Test with Potential to Be Used at Point-of-Care for Pulmonary Tuberculosis: Cape Town Cohort. *J. Mol. Diagn. JMD* **20**, 215–224 (2018).](https://www.zotero.org/google-docs/?54Sgio)

[2. Ushio, R. *et al.* Digital PCR assay detection of circulating Mycobacterium tuberculosis DNA in pulmonary tuberculosis patient plasma. *Tuberc. Edinb. Scotl.* **99**, 47–53 (2016).](https://www.zotero.org/google-docs/?54Sgio)

[3. Click, E. S. *et al.* Detection of Apparent Cell-free M. tuberculosis DNA from Plasma. *Sci. Rep.* **8**, 645 (2018).](https://www.zotero.org/google-docs/?54Sgio)

[4. Cannas, A. *et al.* Mycobacterium tuberculosis DNA detection in soluble fraction of urine from pulmonary tuberculosis patients. *Int. J. Tuberc. Lung Dis. Off. J. Int. Union Tuberc. Lung Dis.* **12**, 146–151 (2008).](https://www.zotero.org/google-docs/?54Sgio)

[5. Fortún, J. *et al.* Extra-pulmonary tuberculosis: differential aspects and role of 16S-rRNA in urine. *Int. J. Tuberc. Lung Dis. Off. J. Int. Union Tuberc. Lung Dis.* **18**, 478–485 (2014).](https://www.zotero.org/google-docs/?54Sgio)

[6. Labugger, I. *et al.* Detection of transrenal DNA for the diagnosis of pulmonary tuberculosis and treatment monitoring. *Infection* **45**, 269–276 (2017).](https://www.zotero.org/google-docs/?54Sgio)

[7. Torrea, G. *et al.* PCR-based detection of the Mycobacterium tuberculosis complex in urine of HIV-infected and uninfected pulmonary and extrapulmonary tuberculosis patients in Burkina Faso. *J. Med. Microbiol.* **54**, 39–44.](https://www.zotero.org/google-docs/?54Sgio)

[8. Rebollo, M. J. *et al.* Blood and urine samples as useful sources for the direct detection of tuberculosis by polymerase chain reaction. *Diagn. Microbiol. Infect. Dis.* **56**, 141–146 (2006).](https://www.zotero.org/google-docs/?54Sgio)

[9. Kafwabulula, M. *et al.* Evaluation of PCR-based methods for the diagnosis of tuberculosis by identification of mycobacterial DNA in urine samples. *Int. J. Tuberc. Lung Dis. Off. J. Int. Union Tuberc. Lung Dis.* **6**, 732–737 (2002).](https://www.zotero.org/google-docs/?54Sgio)

[10. Oreskovic, A. *et al.* Diagnosing Pulmonary Tuberculosis by Using Sequence-Specific Purification of Urine Cell-Free DNA. *J. Clin. Microbiol.* **59**, e00074-21.](https://www.zotero.org/google-docs/?54Sgio)
